# Norwich Medical and Chirurgical School

**Published:** 1831-01-01

**Authors:** 


### LIII.

### NORWICH MEDICAL AND CHIRURGICAL SCHOOL.

WE perceive that a school is formed for the instruction of medical pupils in the great manufacturing city of Norwich, which promises much advantage to the youth of that county who may be preparing for the profession. Anatomy and physiology, we find, are under the superintendence of Dr. Lubbock and Mr. Nichols. The practice of medicine is taught by Dr. England—while the principles, practice, and operations of surgery are ably explained, enforced, and illustrated, by Mr. CROSSE. On Monday, the 4th of October, Mr. Crosse delivered an introductory lecture to a pretty full audience, and was, we believe, well received. We have before us some notes of this introductory lecture; but we need do little more than state, that the matter of the discourse appears to us to be well selected and arranged, though much novelty cannot, of course, be expected on such occasions. Mr. C. has taken an able review of the state of medical education

---

in this and other countries, candidly pointing out the defects of our own institutions, and the excellencies of those in foreign countries. The lecturer, however, gives all due credit to the exertions which have been made of late years, both by corporate bodies and private teachers, to remedy the defects in our systems of medical education, and he reasonably anticipates a still greater amount of amelioration, from the same sources, in the course of a few more years. The beneficial regulations which have recently emanated from the Apothecaries' Company alone are deserving of the highest praise, and have, indeed, extorted approbation from those who are little disposed to accord it to the said body, unless fairly earned.

That company, however, will never have achieved the great object of public utility—which must be theirs also—till they have modified, we will not say annihilated, the system of apprenticeship. Like the College of Surgeons, they ought to enjoin five or six years to be spent in the study of the profession; but they should not insist on more than two, or, at the utmost, three years of INDENTURE, and, consequently, of mere pharmaceutical manipulation. A couple of years is quite sufficient time for the acquisition of pharmacy, as far as the SHOP is concerned—and the other three or four years should be left open to the circumstances or the desires of medical students and their relatives, as to the mode and place of acquiring medical instruction. We conceive that such a modification might be introduced, without all the formalities and expences of parliamentary enactments. In short, the *understanding*, alone, that the indenture of five years is only to be binding for two or three years, would be sufficient to ameliorate the condition of a large class of medical aspirants in this country.

---

P.S. Since the above was written, we find that the Company has actually adopted the modification which we proposed some fifteen months ago. The modification will be seen in the current Number.

